# Exploring the disconnect: mechanisms underpinning the absence of physical function improvement with SGLT2 inhibitors

**DOI:** 10.3389/fsysb.2025.1593229

**Published:** 2025-05-30

**Authors:** Cian Sutcliffe, Jack A. Sargeant, Thomas Yates, Melanie J. Davies, Luke A. Baker

**Affiliations:** ^1^ Department of Respiratory Sciences, College of Life Sciences, University of Leicester, Leicester, United Kingdom; ^2^ Diabetes Research Centre, University of Leicester, Leicester, United Kingdom; ^3^ National Institute for Health Research (NIHR) Leicester Biomedical Research Centre, Leicester, United Kingdom

**Keywords:** SGLT2 (sodium-glucose cotransporter 2) inhibitor, Glucagon-like peptide 1 (GLP-1) receptor agonist, physical function, skeletal muscle, glucose lowering medication, mobility and ageing

## Abstract

Current evidence suggests sodium-glucose cotransporter 2 inhibitors (SGLT2i) do not consistently improve patient physical function, despite improvements in clinical symptoms and reductions in both adiposity and body weight. We highlight heterogenous methodologies in SGLT2i physical function trials. We then provide context to these findings by collating new data which describes how reduced glycaemia with SGLT2i alters numerous physiological processes and discuss how these alterations may diminish or prevent expected functional improvements. Alterations include changes to energy homeostasis, pancreatic hormones, muscle metabolism, physical activity, and appetite regulation. Current evidence in humans is limited and the mechanistic interaction between SGLT2i, skeletal muscle, and physical function remains incompletely understood. Future investigations must embed comprehensive molecular techniques within suitably designed clinical trials to determine how skeletal muscle health and patient mobility is influenced by acute and long term SGLT2i prescription.

## 1 Introduction

Sodium-glucose cotransporter 2 inhibitors (SGLT2i) are approved for the treatment of Type 2 Diabetes (T2DM), chronic kidney disease, heart failure (HF), and long term cardioprotection. SGLT2i drive reductions in body weight via loss of adipose and skeletal muscle tissue (Sargeant et al., 2019). Curiously, despite reductions in body weight, adiposity, and improved cardiac function with SGLT2i, evidence of improved physical function remains equivocal.

Recently, greater emphasis has been placed on the importance of physical function, a predictor of all-cause mortality (Cooper et al., 2010). Despite its clinical significance, it is seldom included as a primary outcome in major trials. Physical function is the ability to perform activities of daily living such as grocery shopping, rather than discretionary exercise- or sport-related tasks. Activities of daily living are underpinned by basic body movements, which in turn are underpinned by the four components of physical fitness (cardiorespiratory fitness, muscle strength, endurance, and flexibility). Physical function assessments vary with respect to accuracy, cost and complexity, yet trials of glucose-lowering therapies (GLT) rarely describe the limitations of the techniques they employ. For example, the cardiopulmonary exercise test (CPET) is a highly accurate assessment of cardiorespiratory fitness, though costly and time consuming. Questionnaires examining physical limitations (e.g., Short Form 36 or Kansas City Cardiomyopathy Questionnaire (KCCQ)) are more convenient for large cohorts but are subjective and poorly correlated with objective measures like CPET and 6-min walk test (6MWT) (Flynn et al., 2012).

On balance, the literature to date shows that SGLT2i do not consistently improve physical function or its determinants. Most research has been performed in HF cohorts where in the CANA-HF trial, Canagliflozin (CANA) did not improve CPET performance (Carbone et al., 2020). Empagliflozin (EMPA) improved CPET performance in a single group of 19 patients with HF and T2DM (Nunez et al., 2018) as well as CPET and 6MWT performance as a secondary outcome in those with HF with reduced ejection fraction (HFrEF) (Santos-Gallego et al., 2021). Larger studies contradict these findings, where EMPA did not improve CPET (Nesti et al., 2022) or 6MWT performance, including the EMPERIAL trials of 627 HF patients with and without T2DM (Abraham et al., 2021). Evidence on dapagliflozin (DAPA) appears ambiguous, with the DETERMINE trials finding no improvement in 6MWT in 313 HFrEF and 504 HFpEF patients taking receiving DAPA (McMurray et al., 2023). However, the PRESERVED-HF trial describes improvements in 6MWT as a secondary outcome among HF patients with preserved ejection fraction (HFpEF) with or without T2DM (Nassif et al., 2021). Physical function tests are not required for the approval of GLT by the US Food and Drug Administration (FDA), therefore, the assessments used in trials are not standardised. Most trials with significant findings have examined function as a secondary outcome variable which are more prone to being statistically underpowered. The validity of certain assessments has been questioned. McMurray et al. (2023) raise the question as to whether SGLT2i truly do not improve physical function or whether the assessments used are in fact poor measures of physical function. Further, changes to physical function have rarely been examined in non-HF cohorts receiving GLT. Designed to address these gaps, the ‘Dapagliflozin, Exercise Training and Physical Function’ (DETA) trial examines whether DAPA improves physical function and whether the addition of structured exercise may augment any benefit. The DETA trial will leverage quantitative, ‘gold standard’ assessments of physical function, which itself is the primary outcome (Sargeant et al., 2024).

Aside from the aforementioned methodological differences, recent mechanistic evidence sheds new light on why SGLT2i may not elicit the expected functional improvements. This evidence describes changes in muscle energy availability, eating behaviours, pancreatic hormones and muscle breakdown. Skeletal muscle mass, endurance and strength are central to physical function, with their importance emphasized in the treatment of T2DM (Strain et al., 2021). Yet, 20%–50% of weight loss with SGLT2i is lean body mass, of which skeletal muscle is the largest component (Sargeant et al., 2019).

Therefore, the aim of this piece is to outline the theoretical basis of SGLT2i’s influence on skeletal muscle and physical function, utilising the latest mechanistic evidence. This newfound understanding may help to explain the equivocal functional outcomes with SGLT2i to date and contextualise the role of physical activity or lifestyle modification as adjuvants to SGLT2i. Debate on this topic is imperative and timely considering the diverse conditions they are now approved to treat and the likely expansion of their regulatory approvals in the future.

## 2 Discussion

### 2.1 Alterations in muscle metabolism

While mice possess the SGLT2 receptor in skeletal muscle (Matthews et al., 2024), humans do not, meaning any effects of SGLT2i on human muscle are assumed to be indirect in nature. Though results from individual studies are highly heterogenous, the mean loss of lean mass as a proportion of total mass with SGLT2i is similar to very low calorie and ketogenic diets (Ashtary-Larky et al., 2022) other pharmacological (including GLP1-ra) and surgical interventions (Neeland et al., 2024). This suggests that the mechanisms we propose in this piece are driven by the loss of glucose (and therefore calories) with SGLT2i. Reduced blood glucose represents a reduction in energy availability for skeletal muscle. However, muscle is an energy-sensitive organ where the regulator of protein synthesis, mammalian target of rapamycin (mTOR) is sensitive to glucose, insulin and amino acid availability (Conn and Qian, 2011). During energy restriction, the key cellular energy sensor AMP-activated protein kinase (AMPK) suppresses energy-consuming processes (e.g., protein synthesis) and enhances energy-producing processes (e.g., glucose and fat oxidation) (Garcia and Shaw, 2017). Therefore, reduced mTOR and enhanced AMPK activity lead to muscle breakdown (Sanchez et al., 2012). In T2DM mice, CANA reduces muscle glucose content by ∼50% while an AMPK activator was three-fold higher than the control group (Nakamura et al., 2023). DAPA suppresses protein synthesis in mouse muscle (Yang et al., 2023) and reduces human skeletal muscle glucose oxidation (Op den Kamp et al., 2022). The rise in circulating ketones observed during SGLT2i therapy supports this energy-deprivation hypothesis, as ketones are produced during energy- and carbohydrate-restriction and can fuel both skeletal and cardiac muscle including in the failing heart (Bedi et al., 2016). We believe these effects are distinct from that of other GLT due to SGLT2i’s unique mechanism of action. Insulin is a highly anabolic hormone that enhances muscle growth and suppresses its breakdown (Schiaffino et al., 2013). It also enhances tissue glucose uptake which improves energy availability within the muscle cell. Other GLT including GLP1-ra, target increased insulin secretion, reduced glucagon secretion, and/or enhanced tissue glucose uptake. In contrast, SGLT2i do not target insulin, glucagon, or tissues. Instead, blood glucose is reduced via urinary excretion leading to lower circulating insulin and reduced energy availability. Further, despite SGLT2i improving insulin sensitivity, the magnitude of improvement is less than the reduction in circulating insulin, meaning a relative reduction in insulin action remains (Merovci et al., 2014).

Thus, there is pre-clinical and clinical evidence that reduced energy availability from SGLT2i may suppress muscle growth by altering cell signalling characteristics. A shift toward pro-catabolic AMPK signalling with SGLT2i has been demonstrated in a number of cell types including kidney (Xu et al., 2021) and cardiac cells (Koyani et al., 2020), though little work has been performed on human skeletal muscle. More research on human muscle is required to determine how enhanced catabolic signalling influences muscle function.

### 2.2 Alterations in pancreatic hormones

Merovci et al. (2014) demonstrated that DAPA was associated with a ∼45% reduction in plasma insulin, ∼30% increase in glucagon and a paradoxical ∼20% increase in endogenous glucose production. These conditions suppress nutrient uptake and its availability within muscle and adipose tissue, instead stimulating lipolysis, glycogenolysis and gluconeogenesis to restore blood glucose. Concerningly, muscle amino acids are used to supply gluconeogenesis via the glucose-alanine cycle, directly linking energy loss with muscle breakdown (Ruderman, 1975). If SGLT2i cause such an outcome, changes in markers of protein breakdown, amino acid transport and muscle mass should be observable. E3 ligases are markers of muscle breakdown and are regulated by forkhead box O (FoxO) transcription factors (Bodine and Baehr, 2014; Kousteni, 2012). CANA-treated mice display significant reductions in mTOR and insulin while E3 ubiquitin ligases and FoxO1 are elevated (Otsuka et al., 2022). FoxO1 also upregulates enzymes involved in gluconeogenesis. In other mice studies where muscle breakdown markers were elevated, so too were the enzymes L-type amino acid transporter 2 (LAT2) and alanine aminotransferase (Otsuka et al., 2022). LAT2 is a membrane-based amino acid transporter while alanine aminotransferase is used to convert amino acids to pyruvate in gluconeogenesis. Pivotally, these mice also experienced reductions in muscle mass and grip strength, but running performance was unchanged (Otsuka et al., 2022). In T2DM mice, muscle content of the amino acids leucine and isoleucine were increased but CANA attenuated these changes (Nakamura et al., 2023). Despite reductions in muscle amino acid content, muscle mass was not reduced which is in contrast to most human studies (Sargeant et al., 2019). In humans, muscle amino acid content is reduced in T2DM (Op den Kamp et al., 2022) and individuals with overweight (Horibe et al., 2022) prescribed DAPA. In the individuals with overweight, muscle loss tended to be greater than control participants though analyses were only performed at 24 weeks by which time body weight (and presumably elevated protein breakdown) had stabilised.

The diuretic effect of SGLT2i also appears to promote muscle breakdown. Despite continuous glucose excretion, diuresis is transient with urine volume returning to baseline within the first few days. Marton et al. (2021) explain in detail how evolutionary mechanisms counteract this diuresis, potentially explaining why it does not persist. Briefly, SGLT2i induce glucose- and sodium-driven osmotic diuresis by perturbing the renal osmotic gradient leading to a loss of energy and fluid. If unchecked, chronic dehydration and loss of energy would pose a threat to the survival of any organism. To prevent this, highly conserved anti-diuretic adaptations occur, whereby muscle amino acids are exploited for nitrogen and energy. This restores the renal osmotic gradient (reference) and energy balance respectively. Nitrogen is required for urea synthesis to restore renal solute equilibrium and conserve water. Though the only sources of nitrogen are dietary protein or endogenous protein stores such as skeletal muscle. Enhanced amino acid catabolism and ureagenesis are evident in those with T2DM and cardiovascular disease receiving EMPA (Kappel et al., 2017). These adaptive processes are energy-intensive, compounding the energy expenditure during glycosuria. Thus, it would follow that compensatory increases in food intake would occur in an attempt to re-establish energy balance (see ‘*Changes in Satiety Hormones and Eating Behaviours*’).

Taken together, available evidence suggests that reduced energy availability and the ensuing alterations in pancreatic hormones caused by SGLT2i stimulate endogenous glucose production that is fuelled in part by muscle protein. If muscle tissue is degraded, potential improvements in physical function may be blunted. Regrettably, current clinical trials lack comprehensive molecular investigations. The US FDA recommends only a representative sample of study subjects should undergo assessment of body composition (Administration US FDA, 2007) with no requirement for assessment of physical function. Therefore, the effect of increased muscle breakdown and the quantity of muscle loss that becomes clinically significant is not known. Detailed mechanistic studies are needed to understand how these changes may impair physical function.

### 2.3 Changes in satiety hormones and eating behaviours

Humans demonstrate substantial metabolic opposition to reduced body weight by increasing energy intake (termed ‘compensatory eating’) and reducing energy expenditure (Rosenbaum et al., 2005), both likely to hinder expected functional improvements. CANA-treated mice display compensatory eating behaviours, increasing food consumption by ∼40%. It is unsurprising that these mice do not lose weight or muscle mass but maintain grip strength. In contrast, CANA-treated mice with fixed food intake experienced reductions in body and muscle mass, with a trend toward reduced running capacity (Otsuka et al., 2022). Macdonald et al. (2022) compared hyperglycaemic mice with and without CANA. Mice given CANA consumed more food than those without. In contrast to most evidence, after 6 weeks of running training the CANA mice improved their run performance while non-CANA mice did not. As this study did not include a pair-fed control group, it is unclear if the beneficial training effects arose from SGLT2i or enhanced energy availability due to compensatory eating. In T2DM participants receiving EMPA, observed weight loss (∼3 kg) was less than predicted (∼11 kg), diverging at 24 weeks (Ferrannini et al., 2015). Energy intake at study termination was ∼270 kcal/day higher than baseline, though this did not prevent loss of lean body mass which was ∼30% of total weight loss. Whether hyperphagia was ineffective in protecting against muscle loss or potentially mitigated even greater reductions is unclear. Unfortunately, physical function was not assessed.

Regarding reduced energy expenditure, CANA-treated mice perform ∼30% less self-selected running and tended to perform worse in a treadmill test (Tanaka et al., 2020), while our group has previously shown a ∼20% reduction in step count among participants receiving EMPA (Yates et al., 2022). Changes in appetite-regulating hormones may explain altered energy intake and expenditure. Leptin chronically regulates energy balance by relaying energy status to the hypothalamus but is reduced by ∼25% during CANA therapy in humans (Ferrannini et al., 2014). Leptin-deficient obese mice are inactive (Coppari et al., 2005) but activity can be restored in a dose-dependent manner via leptin injection (Morton et al., 2011), aligning with patterns observed in humans who have lost weight (Rosenbaum et al., 2002). Ghrelin is a short-acting hormone that drives meal initiation and is ∼50% higher in mice receiving CANA (Naznin et al., 2017), though evidence in humans appears contrasting. Our group examined satiety signals in humans following 24-week EMPA therapy with no observable change to postprandial GLP-1, ghrelin or peptide-YY (Sargeant et al., 2022). Other groups have also found no difference in food intake, energy expenditure, appetite or brain responses to food cues following 12 weeks DAPA (Rajeev et al., 2023).

Increased food intake and potential reductions in self-selected physical activity with SGLT2i is a homeostatic attempt to resolve the negative energy balance caused by glycosuria, gluconeogenesis and urea synthesis. Any functional improvement associated with the lower mechanical burden of decreased bodyweight may be blunted by compensatory eating and/or sedentariness. As lifestyle advice is a cornerstone of any chronic disease treatment, intensive lifestyle counselling is likely needed upon SGLT2i initiation to counteract these unfavourable changes.

Whether different medications within the SGLT2i class, or SGLT2i as part of combination therapy exert differential effects is not known. The SGLT2:SGLT1 receptor selectivity of CANA is ∼250:1, while DAPA (∼1,200:1) and EMPA (∼2,500:1) are more specific to SGLT2. SGLT1 is responsible for the majority of intestinal glucose uptake and is needed to enhance insulin release and inhibition of glucagon secretion after glucose consumption (the ‘incretin effect’) (Gorboulev et al., 2012). In theory, greater SGLT1 inhibition would exacerbate reduced energy availability, though this has not yet been confirmed. Future evidence from the recently approved Sotagliflozin, a non-specific SGLT2i (∼20:1) will be of great interest.

### 2.4 Changes in cytokines and systemic inflammation

Systemic inflammation is commonly observed in chronic conditions (Furman et al., 2019) with elevated Interleukin 6 (IL-6) and Tumour Necrosis Factor-α (TNF-α) predictive of T2DM development (Spranger et al., 2003). However, inflammatory cytokines also promote skeletal muscle breakdown by suppressing protein synthesis and enhancing breakdown, which may compromise physical function. TNF-α impairs activation of AKT (de Alvaro et al., 2004), a key player in the mTOR protein synthesis pathway, while indirectly enhancing E3 ligase transcription (Cai et al., 2004). IL-6 can indirectly activate Myostatin, the negative regulator of muscle growth (Zhang et al., 2013). Finally, insulin-like growth factor 1 (IGF-1) is highly anabolic, blunting E3 ligase expression and stimulating mTOR, though IL-6 reduces serum IGF-1 (Nemet et al., 2006). As weight loss interventions reduce pro-inflammatory markers (Ouchi et al., 2011), SGLT2i modestly reduce systemic inflammation (Bray et al., 2020), though some results are discordant. Koyani et al. (2020) used Lipopolysaccharide (LPS) to induce an acute inflammatory response in cardiomyocytes, which showed enhanced TNF-α expression from 1 to 8 h. Cells pre-treated with a physiological dose of EMPA showed a blunted inflammatory response. The authors repeated this in rats, where circulating TNF-α and creatine kinase, a marker of muscle damage, were significantly reduced. However, the observation in rodents occurred following an acute (8-hour) exposure to a dose 20–50 times higher than used in humans. Given the interrelated multi-system changes induced by SGLT2i, the extent to which acute exposure to SGLT2i and inflammation accurately represents a patient on multi-year SGLT2i therapy is limited.

In humans, examination of inflammatory cytokines among 200 individuals with T2DM receiving CANA yielded conflicting results with serum TNF-α increasing by 7% while IL-6 decreased by 22% (Garvey et al., 2018). Unfortunately, changes were only examined after 52 weeks. Future studies assessing transient changes to inflammatory markers that might modulate muscle atrophy in the short term likely require more frequent measurement. SGLT2 inhibitors anti-inflammatory actions should in theory protect against reductions in muscle mass and function, though there is a dearth of long-term trials examining inflammation, muscle and physical function in tandem. At present, it would be premature to hold any definitive stance on a SGLT2i-inflammation-atrophy axis. More *in-vitro* and skeletal muscle-specific investigations are required, as any systemic anti-inflammatory effects of SGLT2i must be realised at the muscle tissue level before any benefits can be attained.

### 2.5 Conclusion

**FIGURE 1:**
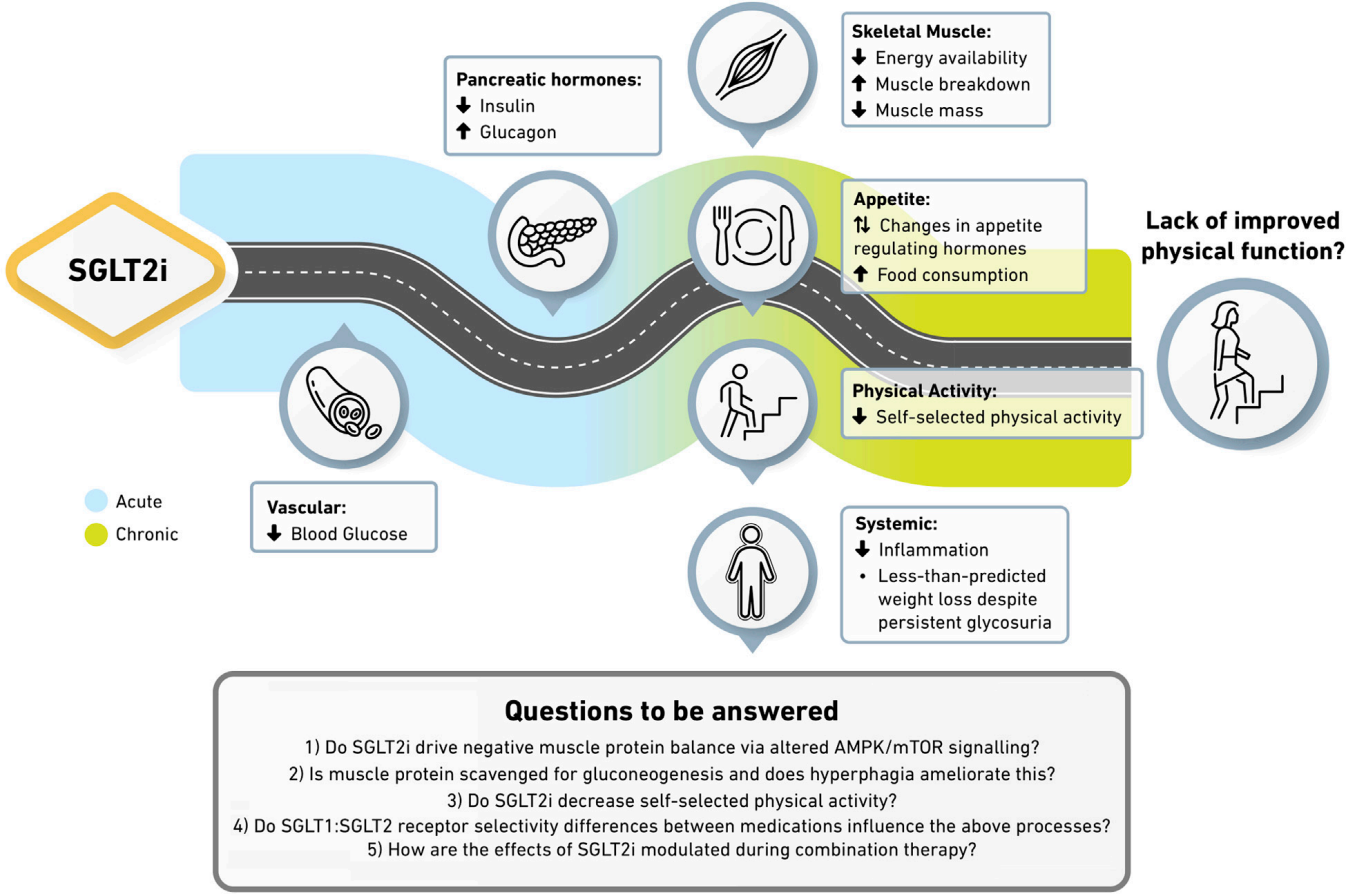
Potential mechanisms underpinning the absence of physical function improvement with SGLT2i. SGLT2i reduce blood glucose while reducing the insulin to glucagon ratio. Reduced energy availability and anabolic signalling within skeletal muscle drives enhanced protein breakdown and reductions in muscle mass. Altered satiety hormones stimulate greater food intake and sedentary behaviours, blunting weight loss despite continued glycosuria. More suitably powered trials underpinned by comprehensive molecular techniques are required to confirm this hypothesis. In particular, how SGLT1:SGLT2 receptor selectivity differences between medications and how SGLT2i in combination with other therapies modulate these processes remains unknown.

To conclude, whether SGLT2i influence physical function is not fully clear, though reduced energy availability, increased muscle breakdown, and unfavourable changes to diet or physical activity may blunt potential improvements. With SGLT2i and GLP1 receptor agonist prescription increasing rapidly, it is timely that their secondary effects are understood in order to provide optimal patient care. At present, the risk/benefit balance of SGLT2i is unquestionably favourable and we do not dismiss their demonstrable benefits. Instead, we draw attention to potentially unfavourable side effects which may warrant some caution, particularly for those with or at high risk of sarcopenia. This may be of greater concern with GLP1 receptor agonists given the larger degree of weight loss (Figure 1). Gaps in available evidence leave the following questions unanswered; 1) do SGLT2i stimulate negative muscle protein balance via altered AMPK/mTOR signalling? 2) is muscle protein scavenged for gluconeogenesis and does hyperphagia ameliorate this? 3) do SGLT2i decrease self-selected physical activity? 4) do intra-class SGLT1:SGLT2 receptor selectivity differences influence the above processes? 5) How are the effects of SGLT2i modulated during combination therapy? To address these questions and provide the holistic understanding needed to inform clinical practice, trials powered to detect a change in physical function should include groups with fixed food intake, while also frequently assessing muscle metabolism and satiety hormones in the acute phase. As age, exercise, nutrition and the severity of disease contribute to the rate of muscle decline, if SGLT2i drive maladaptation within skeletal muscle then these personal characteristics may need to be considered when deciding the most appropriate pharmacotherapy.

## Data Availability

The original contributions presented in the study are included in the article/supplementary material, further inquiries can be directed to the corresponding author.
